# Geminal-Based Strategies for Modeling Large Building Blocks of Organic Electronic Materials

**DOI:** 10.1021/acs.jpclett.3c02434

**Published:** 2023-10-30

**Authors:** Paweł Tecmer, Marta Gałyńska, Lena Szczuczko, Katharina Boguslawski

**Affiliations:** Institute of Physics, Faculty of Physics, Astronomy, and Informatics, Nicolaus Copernicus University in Toruń, Grudziadzka 5, 87-100 Toruń, Poland

## Abstract

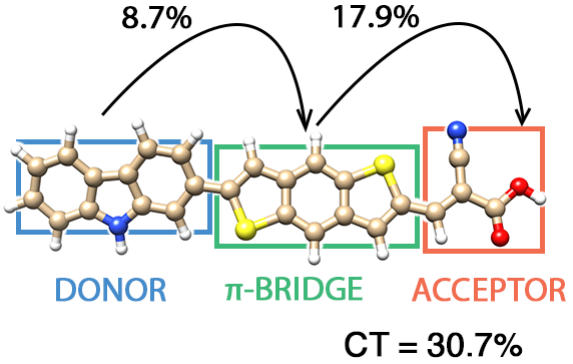

We elaborate on unconventional electronic structure methods based on geminals and their potential to advance the rapidly developing field of organic photovoltaics (OPVs). Specifically, we focus on the computational advantages of geminal-based methods over standard approaches and identify the critical aspects of OPV development. Examples are reliable and efficient computations of orbital energies, electronic spectra, and van der Waals interactions. Geminal-based models can also be combined with quantum embedding techniques and a quantum information analysis of orbital interactions to gain a fundamental understanding of the electronic structures and properties of realistic OPV building blocks. Furthermore, other organic components present in, for instance, dye-sensitized solar cells (DSSCs) represent another promising scope of application. Finally, we provide numerical examples predicting the properties of a small building block of OPV components and two carbazole-based dyes proposed as possible DSSC sensitizers.

Organic photovoltaic (OPV) devices and dye-sensitized solar cells (DSSC) represent an up-and-coming technology. For instance, their ever-improving^1^ cell efficiency (over 19%)^2^ and performance lifetime, combined with low environmental impact and potential roll-to-roll manufacturing, make OPVs competitive with conventional silicon-based technologies. An additional advantage of OPV-based materials is the diversity of organic molecules that can be used to design building blocks of donors, acceptors, and their interfaces. Unfortunately, the experimental search for optimal OPV components is costly and very time-consuming. Thus, reliable quantum chemical methods combined with an efficient and flexible software package design are of utmost importance in the search for new, more efficient building blocks of OPV materials.^3,4^ Commonly available models, like Density Functional Approximations (DFAs),^5,6^ do not always provide reliable results for large extended π-systems,^7^ common building blocks of OPVs,^8^ and lack systematic improvability.^3,9^ While semilocal and global hybrid exchange–correlation functionals tend to overestimate the delocalization of electron and hole densities, the range-separated hybrids tend to overlocalize electron densities in π-conjugated chains.^8,10−12^ As a consequence, the DFAs lead to an underestimation of torsional barriers, an overestimation of the bond-length alternation in polyenes, and incorrect band gaps.^13,14^ Another difficulty originates from their electronic structures’ biradical or multireference nature, which is often remarkably challenging to describe within a single-reference framework. Moreover, the standard exchange–correlation functionals tend to suffer in predicting charge transfer excitation energies.^15^ At the same time, the applicability of standard wave function-based method is very limited, mainly due to unfavorable computational scaling of multireference approaches. Geminal-based methods are a promising alternative to conventional quantum chemistry models, providing a more compact representation of the correlated wave function.^16−27^ Commonly used examples are the wave function classes based on the Antisymmetric Product of 1-reference orbital Geminal (AP1roG),^28,29^ also known as pair Coupled Cluster Doubles (pCCD),^30^ the Antisymmetrized Product of Strongly orthogonal Geminals (APSG),^31^ the Generalized Valence Bond (GVB),^32^ and their orbital optimized variants (for a recent review, see ref (19)).^29,33−36^ Combined with a reliable a posteriori correction to account for the missing dynamic correlation effects,^37−48^ they allow us to model electron correlation effects effectively and in a balanced way.^49^ Except for perturbation-based approaches, which might break size-extensivity, these geminal-based models can be upscaled to model OPV-devices. On top of that, some further methodological developments are indispensable to meet the requirements of OPV applications and their transferability to other organic-based electronic molecules like those encountered in DSSCs. Below, we list the key challenges that geminal-based methods must overcome to become a potential driving force in the design of modern organic electronic building blocks and demonstrate some initial numerical examples. Our focus is set on quantum chemical models that could be upscaled to large systems and provide reliable results for OPV-related properties.

## Orbital Energies

OPVs can, in general, generate electricity from sunlight if the energy of light is equal to or greater than the donor–acceptor band gap offset. Thus, a critical factor in designing novel organic-based donor and acceptor molecules is the knowledge of the energies of the Highest Occupied Molecular Orbital (HOMO) and the Lowest Unoccupied Molecular Orbital (LUMO) and the corresponding HOMO–LUMO gap. While these are easily obtained from the Hartree–Fock or DFA-based methods employing Koopman’s or Janak’s theorem,^50^ respectively, they are not always reliable. Furthermore, Janak’s theorem is valid only for LDA and GGA-type exchange–correlation functionals and not for the generalized Kohn–Sham methods. The latter might suffer from incorrect treatment of fractionally normalized charges.^10−12,51^ Moreover, the LUMO energy shows very little correlation with the electron affinity.^14^ On the other hand, most geminal theories work with natural orbitals, where a (natural) occupation number is associated with each orbital, and orbital energies are not directly available. They must be deduced from the existing wave function, simultaneously ensuring efficiency and accuracy. One of the simplest approximations uses information about the diagonal elements of the Fock matrix and the electron repulsion energy.^52^ More reliable orbital energies can be obtained from the ionization potential (IP)^53−56^ and electron affinity (EA)^57^ variants of Equation-of-Motion (EOM)^58−60^ applied on top of a geminal reference wave function.^61^ From the IPs and EAs, we can deduce what is called the charge gap in the solid-state community (also referred to as the fundamental gap or HOMO–LUMO gap)
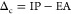
1However, recent benchmark studies using the IP-EOM-pCCD approach^62^ unravel the importance of dynamic correlation energy in reproducing reference data for small, compact organic molecules. Thus, we require further methodological development and steeper computational scaling. The remedy seems to be deriving and calculating orbital energies from the extended Koopman’s theorem (EKT) on top of orbital-optimized methods.^63−65^ The approximate orbital energies are then determined by solving the secular equation
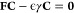
2where **C** is the matrix of eigenvectors, ϵ is the diagonal matrix of eigenvalues (orbital energies), γ_*pq*_ is the one-particle reduced density matrix (1-RDM)
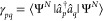
3and **F** is the so-called generalized Fock matrix (aka Lagrangian)
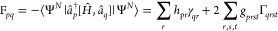
4which can be expressed in terms of one- and two-electron integrals, *h*_*pr*_ and *g*_*prst*_, and reduced density matrices. In the above expression, the two-particle reduced density matrix (2-RDM) is defined as
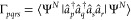
5Thus, to calculate (approximate) orbital energies from orbital-optimized geminal-based methods, the corresponding 1- and 2-RDMs must be reliable. An alternative formulation of EKT, proposed by Ciosłowski and co-workers,^66^ uses energy-derivative density matrices.

## Electronic Properties

The power conversion efficiency of OPV devices can be improved by exploiting building blocks that feature a complementary spectral absorption range between the donor and the acceptor and a strong absorption in the visible-near-infrared region to ensure a large short-circuit current. To predict such properties through large-scale quantum chemical modeling, we must efficiently and reliably determine the electronic spectra (electronic excitation energies and associated transition dipole moments) and ground-state electronic properties, such as electronic dipole and quadrupole moments. Optimizing these properties is indispensable for improving the charge separation, transport, and overall device performance in organic solar cells. Having computed the lowest excitation energy, also denoted as the optical gap (Δ_o_), and the charge gap (eq 1), we can determine the exciton binding energy^67^
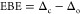
6The EBE denotes the energy required to dissociate an excited electron–hole pair into free charge carriers. Specifically, the exciton is formed in the donor domain of the OSC. At the same time, the acceptor material is meant to provide a way to overcome the corresponding EBE and hence separate the charges. Thus, we should be able to predict reliable EBEs for the donor and the donor–acceptor interface to steer the efficiency of OPV devices.

Most promising geminal theories for excited-state calculations are based on the extended random phase approximation^39,68^ and the EOM formalism.^69−72^ Although most EOM-based methods yield size-intensive energies, the corresponding properties derived from transition density matrices are not. Yet, the computations of transition dipole moments from EOM could be more computationally impractical due to the need to compute both left and right eigenvectors. A remedy to this problem is linear-response theory, which can be used in large-scale modeling.^73^ Finally, we stress that a reliable prediction of electronic dipole and quadrupole moments from geminal theories requires the inclusion of single excitations in the theoretical model.

## Noncovalent Interactions

The materials of the OPV’s active layer should exhibit suitable aggregation properties to form nanoscale phase separations and interpenetrating networks. Thus, quantum chemistry methods must accurately predict the intermolecular interaction energies of large molecules. Such a task is remarkably difficult because the interaction energy often features a considerable amount of noncovalent/dispersion interactions, which are challenging to model reliably employing methods designed for strong electron correlation. An exception are geminal-based approaches, which proved to be reliable and computationally efficient in describing systems featuring a mixture of nondynamic and noncovalent interactions.^46,47,73−78^ Apart from Symmetry Adapted Perturbation Theory^79^ and a linearized coupled-cluster correction on top of a geminal reference function,^77,80^ new geminal-based models are highly desirable to facilitate modeling of noncovalent interactions and large molecules. A promising approach to theoretically describe large-scale noncovalent interactions in OPVs is a hybrid method that combines a given geminal ansatz with a semiclassical dispersion correction. In such models, the geminal part captures long-range electron correlation effects, while short-range dynamic electron correlation is handled by DFAs and a semiclassical dispersion correction to account for long-range dynamic correlations.^81^ Furthermore, the commonly used exchange–correlation functionals can be combined with a D3 dispersion correction and Becke–Johnson damping.^82^

## Quantum Embedding with Geminals

Another challenge the quantum chemical modeling of realistic OPVs faces is the need to cope with many atoms. A promising approach to circumvent this problem and reduce the computational cost dramatically originates from quantum embedding techniques.^83^ Its idea relies on the fact that electron correlation is “*local*” in nature,^84,85^ allowing us to partition the whole system into subsets.^86,87^ Examples are the WFT-in-DFT and WFT-in-WFT approaches, where a geminal wave function treats only a small subset of atoms in the whole molecular structure. In contrast, the remaining part is treated with a computationally more efficient, albeit less accurate, method. Such approaches effectively account for environmental effects present in OPVs. So far, only the simplest static embedding model has been tested for geminals.^88^ More reliable embedding schemes exploiting orbital optimization procedures within geminals, similar to the “*freeze-and-thaw*” protocol,^89^ are yet to be developed.

## Quantum Entanglement and Correlation

Due to the restriction to electron-pair excitations, the corresponding 1- and 2-RDMs can be calculated computationally more effectively than in conventional wave function-based methods. The approximate 1- and 2-RDMs obtained from (orbital-optimized) geminal-based wave functions^25,27,49,90−92^ can be used to calculate the single- and two-orbital entropies from which quantum entanglement and correlation measures can be computed.^90,91,93−97^ The single-orbital entropy determines the quantum entanglement between each orbital and the orbital bath and can be applied to deduce molecular bond-orders^95,98,99^ and guide the partitioning of the quantum system.^88,90,100−102^ The so-called mutual information allows us to dissect electron correlation into different types^94^ and quantify the interaction between orbitals in the system (in terms of orbital-pair correlations).^100,103^ Altogether, these tools provide a deeper understanding of electronic structures by using the language of interacting orbitals. Being able to scrutinize the interactions of the HOMO and LUMO orbitals with the remaining molecular orbitals might guide the optimization process for developing new organic solar cells and their building blocks.

## Interpretational Potential

Since geminal-based methods exploit two-electron functions as the fundamental building blocks of the electronic wave function, the corresponding molecular orbital basis (used to construct each geminal) is typically localized on just a few centers of the whole molecule. Due to these localized molecular orbitals, the contributions to excited, ionized, or electron-attached states feature several contributions (Slater determinants) with small weights. Thus, the underlying electronic structure differs from the conventional picture we obtain when working with delocalized canonical orbitals such as those predicted by DFAs. The significant advantage of a localized basis^104,105^ is the clear distinction of the donor and acceptor regions or their interfaces.^106^ Electronic excitations can be unambiguously assigned to specific molecular basins, allowing dissection of the electronic excitations into, for instance, charge transfer or local ones, while HOMO and LUMO orbitals can be located on, for instance, donor or acceptor domains. Such an analysis is particularly beneficial in designing OPV building blocks that are desired to feature HOMO/LUMO on specified domains or increase the charge-transfer character in the excited states of interest.

## Interoperable and Reusable Software

A key factor in developing new methods, like geminals, is their implementation in modern software packages, fulfilling the desired FAIR (Findable, Accessible, Interoperable, and Reusable) features.^107^ While many geminal methods are scattered across different software platforms and packages,^73,108−110^ the interoperability and reusability of geminal-based software are indispensable for their faster development, testing, and application to OPV-related problems. Geminal-based methods could become a driving force for quantum chemical modeling of organic electronic compounds or their building blocks if such requirements are genuinely fulfilled.

## Numerical Examples

**Figure 1:**
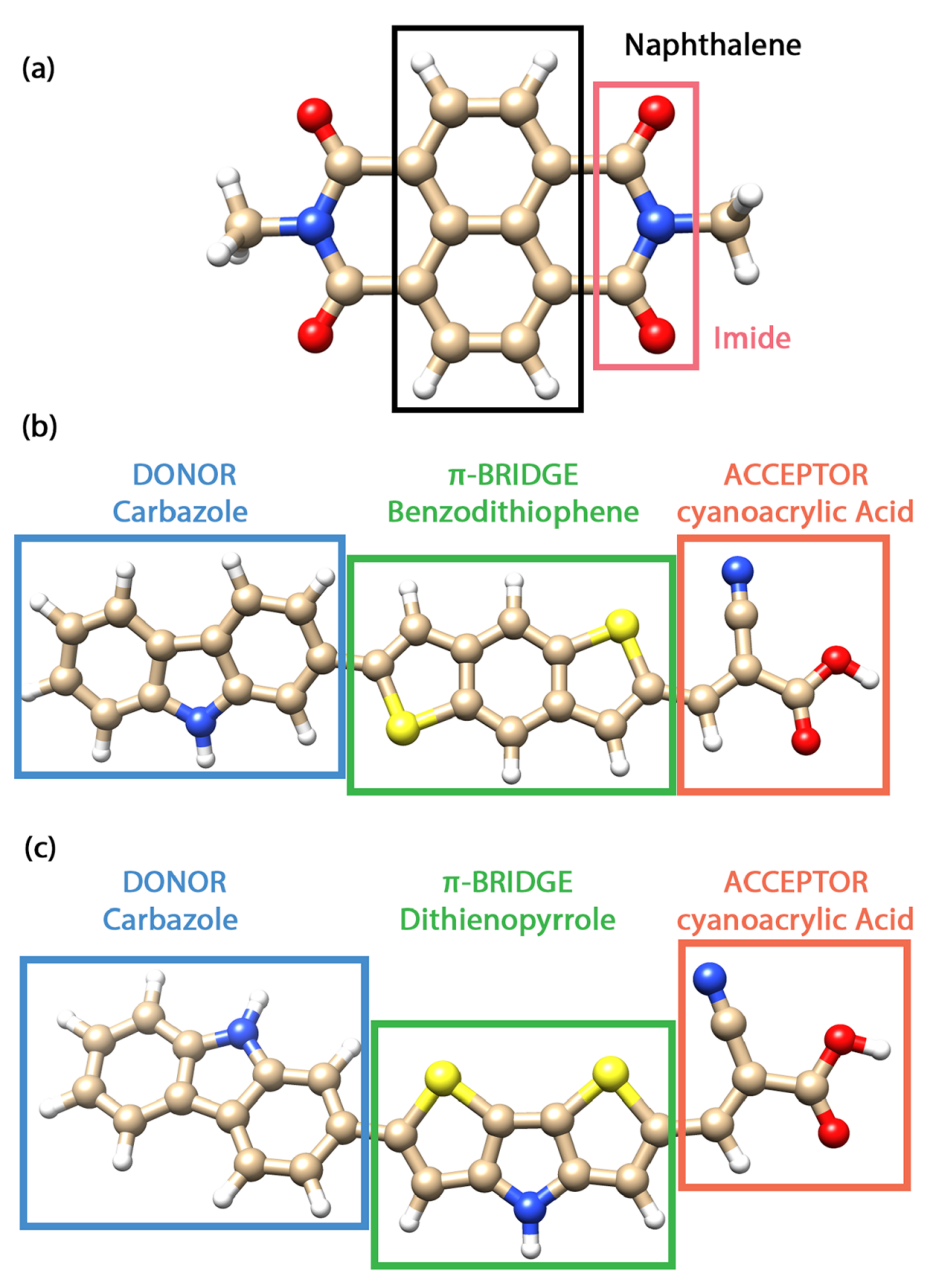
Molecular structures of (a) naphthalene diimide (NDI), two carbazole-based dyes with different π-bridge units such as (b) benzodithiophene (CBA) and (c) dithienopyrrole (CDA). For structures (b) and (c), the donor, π-bridge, and acceptor components are highlighted with blue, green, and red rectangles, respectively. Structure (a) was relaxed using B3LYP/cc-pVDZ, while (b) and (c) were optimized using M06/6-31G(d), and their geometries are available in the Supporting Information of ref (111).

In the following, we illustrate the performance of orbital-optimized pCCD methods in predicting various molecular properties in three organic molecules, depicted in Figure 1. These molecules represent examples of how to advance the performance of organic electronic components: naphthalene diimide (NDI) as an excellent acceptor (see Figure 1a) and two carbazole-based dyes of DSSCs proposed in ref (111), namely CBA (see Figure 1b) and CDA (see Figure 1c), respectively. The electronic properties of those molecules in terms of the HOMO and LUMO energies, IP and EA, and Δ_c_ calculated from the IP/EA-EOM-pCCD models using the 1h/1p (1 hole/1 particle) and 2h1p/2p1h (2 holes 1 particle/2 particles 1 hole) operators are presented in Table 1. For NDI, we compare the pCCD results to the Restricted Hartree–Fock (RHF), EOM-DLPNO-CCSD, B3LYP,^112^ and experimental results. For the carbazole-based dyes, the performance of pCCD is compared to PBE^113,114^ (GGA functional), PBE0^115^ (hybrid functional with 25% of HF exchange), CAM-B3LYP^116^ (range-separated hybrid exchange–correlation functional with 19% and 65% of HF exchange for the short and long-range, respectively), the statistical average of orbital model potential (SAOP),^117^ and IP/EA-EOM-DLPNO-CCSD.^118^

**Table 1:** Performance of Various Quantum Chemistry Methods in Predicting HOMO, LUMO, and Δ_c_ Energies (in eV)

	HOMO	LUMO	Δ_c_
**NDI**			
B3LYP/6-311g(d, p)^124^	–7.25	–3.61	3.64
RHF/cc-pVDZ	–9.21	–0.41	8.80
IP/EA-EOM-DLPNO-CCSD/cc-pVDZ	–8.78	–1.51	7.27
IP/EA-EOM-pCCD(1h/1p)/cc-pVDZ	–9.89	0.42	10.31
IP/EA-EOM-pCCD(2h1p/2p1h)/cc-pVDZ	–6.85	–3.11	3.74
Experimental^124^	–6.78	–3.63	3.15
**CBA**			
PBE/TZ2P	–5.46	–3.72	1.74
PBE0/TZ2P	–6.23	–3.07	3.16
CAM-B3LYP/TZ2P	–7.20	–2.26	4.94
SAOP/TZ2P	–9.51	–1.69	7.82
IP/EA-EOM-DLPNO-CCSD/cc-pVDZ	–7.07	–1.02	6.05
IP/EA-EOM-pCCD(1h/1p)/cc-pVDZ	–7.86	0.90	8.76
IP/EA-EOM-pCCD(2h1p/2p1h)/cc-pVDZ	–4.91	–2.59	2.32
**CDA**			
PBE/TZ2P	–5.27	–3.47	1.80
PBE0/TZ2P	–6.00	–2.94	3.06
CAM-B3LYP/TZ2P	–6.94	–2.06	4.88
SAOP/TZ2P	–9.23	–7.45	1.78
IP/EA-EOM-DLPNO-CCSD/cc-pVDZ	–6.66	–0.80	5.86
IP/EA-EOM-pCCD(1h/1p)/cc-pVDZ	–7.51	1.05	8.56
IP/EA-EOM-pCCD(2h1p/2p1h)/cc-pVDZ	–4.51	–2.40	2.11

NDI, a member of the rylene diimide molecule class,^119−122^ is composed of a naphthalene core—a conjugated π system—linked at both termini to imide units (Figure 1a). NDI and its derivatives showcase excellent n-type semiconductor characteristics, rendering them suitable acceptors in organic solar cells (OSCs) due to their robust electron acceptor properties, high chemical and thermal stability, substantial absorption coefficient, and prominent fluorescence.^123^ Theoretical investigations^124^ demonstrated that the B3LYP functional yields HOMO and LUMO energies in good alignment with experimental values. The overestimation of the HOMO energy by 0.47 eV leads to a larger HOMO–LUMO gap of 0.49 eV compared with experimental findings. Significantly poorer results emerged with the RHF method, which decreases the HOMO energy by 1.96 eV and increases the LUMO energy by 3.2 eV relative to the B3LYP results. This shift produces a massive Δ_c_ energy of 8.80 eV. IP/EA-EOM-DLPNO-CCSD only slightly corrects the HOMO and LUMO energies, deviating by about 2 eV from the experimental results, while the error in the charge gap increases to 4 eV. IP/EA-EOM-pCCD(1h/1p) yields even more significant HOMO and LUMO energy errors. If the IP/EA description is treated more accurately and extended to the 2h1p/2p1h level, the HOMO energy aligns excellently with experimental results (within chemical accuracy), while the LUMO is underestimated by 0.52 eV. The larger error of the LUMO level increases the Δ_c_ gap by 0.59 eV compared to experiment. Note that the EA-EOM formalism is more sensitive to the basis set size and environmental effects;^125^ hence, larger errors in LUMO energies are expected.

**Figure 2:**
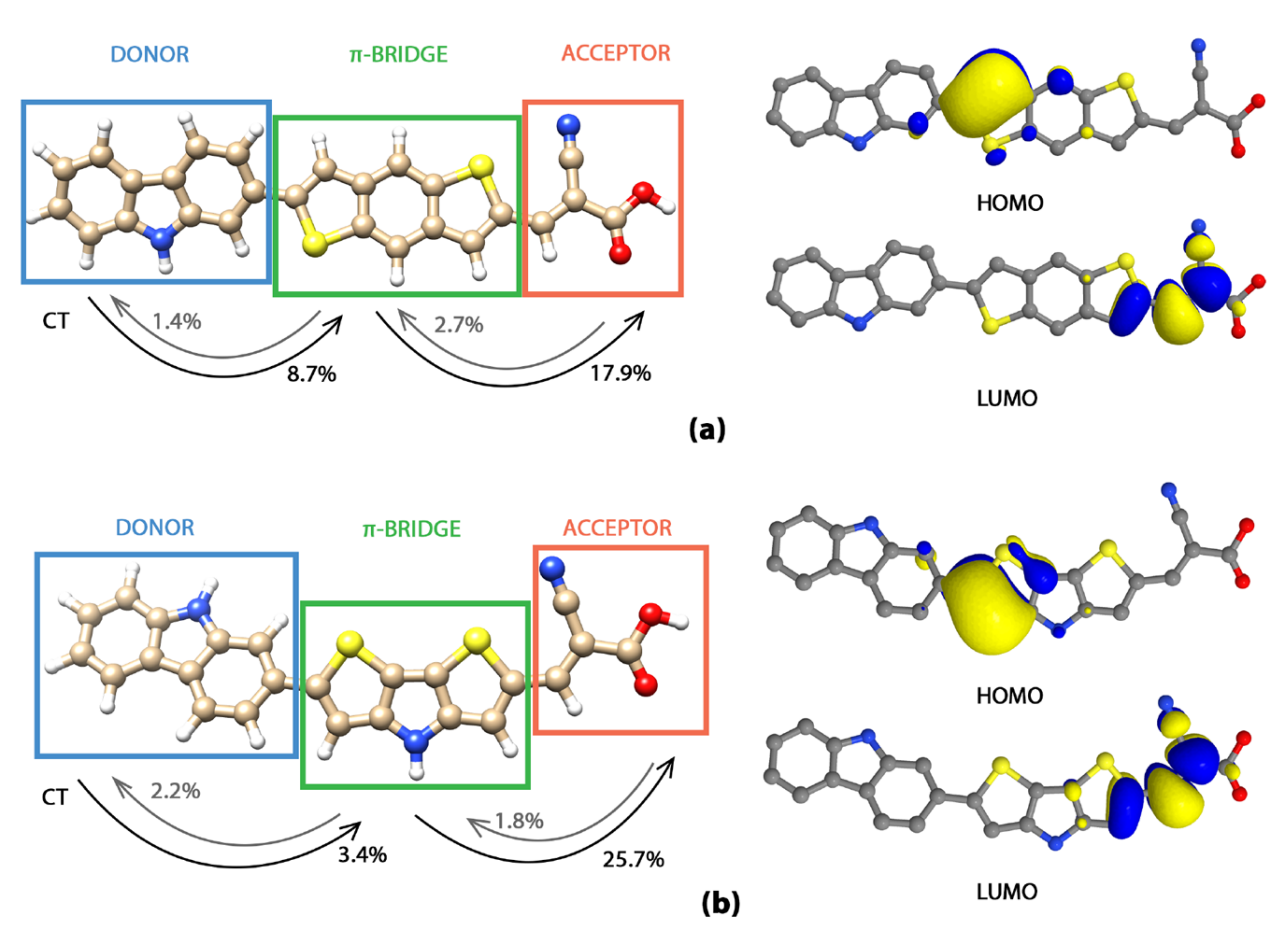
pCCD HOMO and LUMO orbitals of the (a) CBA and (b) CDA dyes. On the left-hand side, the percentage of charge transfer character to the lowest-lying excited state for each dye is included.

The two carbozyle-based dyes shown in Figure 1b,c were proposed in ref (111) as new donor−π-bridge–acceptor organic sensitizers for DSSCs. These dyes incorporate a carbazole donor and a cyanoacrylic acid acceptor while utilizing benzodithiophene and dithienopyrrole as the π-spacers in CBA and CDA, respectively. Accurately determining the energy alignment between a sensitizer and a semiconductor substrate (such as TiO_2_) is pivotal in assessing the applicability of a dye in DSSCs. Hence, precise determination of the HOMO and LUMO energies is paramount. As indicated in Table 1, the HOMO, LUMO, and Δ_c_ energies strongly rely on the fraction of HF exchange and the choice of the DFA. For instance, for the CBA dye, the HOMO and LUMO energies vary by up to 4.05 and 2.03 eV, respectively. Such substantial discrepancies in the frontier orbital energies notably affect Δ_c_, ranging from 1.74 to 7.82 eV. Like NDI, Δ_c_ determined by IP/EA-EOM-pCCD(1h/1p) is significantly reduced when the 2h1p/2p1h sectors are employed in the IP/EA models. Furthermore, as observed for NDI, IP/EA-EOM-DLPNO-CCSD predicts a charge gap more than twice as large as IP/EA-EOM-pCCD(2h1p/2p1h). Finally, we should note that the localized nature of the pCCD-optimized orbitals allows us to locate the HOMO and LUMO across the dye sensitizer: while the LUMO of both CBA and CDA is located on the acceptor domain, the HOMO is centered mainly on the π bridge (see Figure 2).

In contrast to the frontier orbital energies, the discrepancies in the lowest-lying excited state energies are much less pronounced if DFAs are concerned, differing by a maximum of 1.07 eV across the investigated functionals (2.08/2.32 for PBE, 2.66/2.75 for PBE0, 3.11/2.88 for CAM-B3LYP, and 2.04/2.23 for SAOP for CBA/CDA, respectively). The first excitation energy further increases in EOM-pCCD+S calculations to 4.76 eV for CBA and 4.36 eV for CDA, respectively. A detailed analysis of the excited state wave function at the EOM-pCCD+S level of theory, considering about 90% of the configurational weights, suggests that the lowest-lying excited state of the CBA dye features around 30% charge transfer character, involving electronic transitions between donor and acceptor units, from which the majority (87%) entails charge transfer from the donor to the acceptor unit (Figure 2a). In contrast, the charge transfer character in the CDA molecule increases to 33%, with a similar portion of 88% going from the donor to the acceptor domain (Figure 2b). A similar analysis, considering only 71% (CBA) or 74% (CDA) of the configurational space, is included in the Supporting Information.

Thus, our numerical examples illustrate that pCCD-based methods can predict accurate molecular properties (HOMO/LUMO energies and charge/band/fundamental gaps). In contrast, the localized nature of the molecular orbital basis allows us to unambiguously identify the domain on which the HOMO or LUMO orbitals are centered. Furthermore, each excited state wave function can be broken down into electronic excitations centered on specific domains such as local or CT excitations. Although such an analysis might initially seem tedious, it is possible to fully automate the assessment of the excitation characters by assigning molecular orbitals to specific domains. Finally, another challenge of geminal-based methods will be pushing the precision to chemical accuracy, considering environmental effects or dynamical correlation.

## Supporting Information Available

Excitation spectra and their contributions (Figures S1–S6) and computational details (PDF)
