# Supplementary material for: Geminal-Based Strategies for Modeling Large Building Blocks of Organic Electronic Materials
Source: J Phys Chem Lett. 2023 Oct 30;14(44):9909–17. doi: 10.1021/acs.jpclett.3c02434 (PMC10641881; doi:10.1021/acs.jpclett.3c02434)
Supplement: Supplementary file 1 — jz3c02434_si_001.pdf [file jz3c02434_si_001.pdf]

Geminal-based strategies for modeling large building blocks of organic electronic materials

Paweł Tecmer, Marta Gałyńska, Lena Szczuczko, and Katharina Boguslawski

Institute of Physics, Faculty of Physics, Astronomy and Informatics,
Nicolaus Copernicus University in Torun,
Grudziadzka 5, 87-100 Torun, Poland

Supplementary Information

S1 Excitation spectra and their contributions

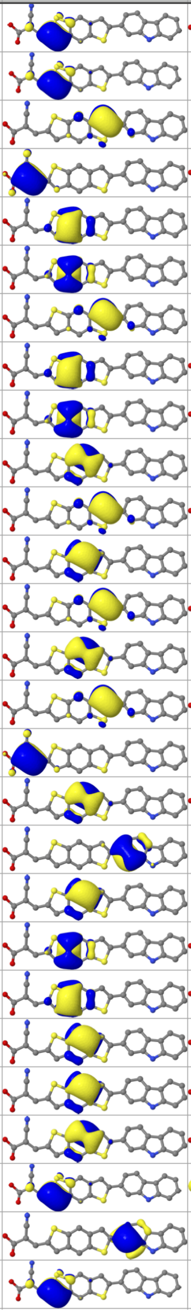
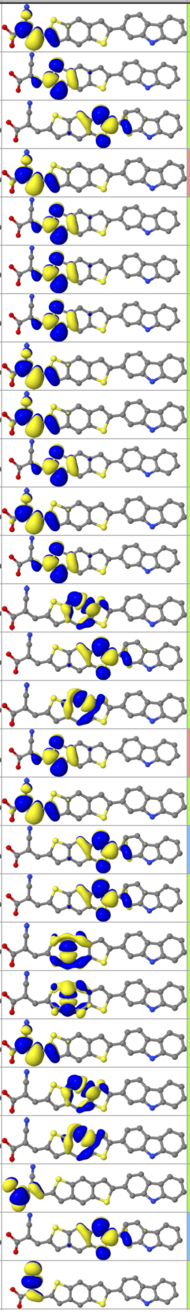

Initial	Final	CI	|CI|²	Initial MO	Final MO	Initial MO type	Final MO type
113	121	0.26755	0.07158			B-pi	A-pi*
113	120	0.25090	0.06295			B-pi	B-pi*
116	118	0.23292	0.05425			B-pi	B-pi*
112	121	-0.19582	0.03835			A-pi	A-pi*
87	120	0.15988	0.02556			B-pi	B-pi*
86	120	-0.15772	0.02488			B-pi	B-pi*
116	120	0.13591	0.01847			B-pi	B-pi*
87	121	0.12625	0.01594			B-pi	A-pi*
86	121	-0.12458	0.01552			B-pi	A-pi*
89	120	-0.12245	0.01499			B-pi	B-pi*
116	121	0.11607	0.01347			B-pi	A-pi*
85	120	-0.11551	0.01334			B-pi	B-pi*
116	135	-0.11482	0.01318			B-pi	B-pi*
89	118	-0.10797	0.01166			B-pi	B-pi*
116	146	-0.10650	0.01134			B-pi	B-pi*
112	120	-0.10329	0.01067			A-pi	B-pi*
89	121	-0.10280	0.01057			B-pi	A-pi*
94	118	0.10093	0.01019			D-pi	B-pi*
85	118	-0.10043	0.01009			B-pi	B-pi*
86	144	0.09832	0.00967			B-pi	B-pi*
87	145	0.09795	0.00959			B-pi	B-pi*
85	121	-0.09702	0.00941			B-pi	A-pi*
85	135	0.09695	0.00940			B-pi	B-pi*
89	146	0.09682	0.00937			B-pi	B-pi*
113	122	0.09414	0.00886			B-pi	A-pi*
82	118	0.09393	0.00882			D-pi	B-pi*
113	119	0.09316	0.00868			B-pi	A-pi*

Figure S1: Dominant contributions to the first excited state in benzodithiophene (CBA). The excitation goes from the initial to the final MO, whose isosurface and type are indicated in the last four columns. Only contributions with $|CI| > 0.05$ are shown. Donor MOs are labeled in blue, acceptor MOs in red, while green indicates MOs centered on the bridge.

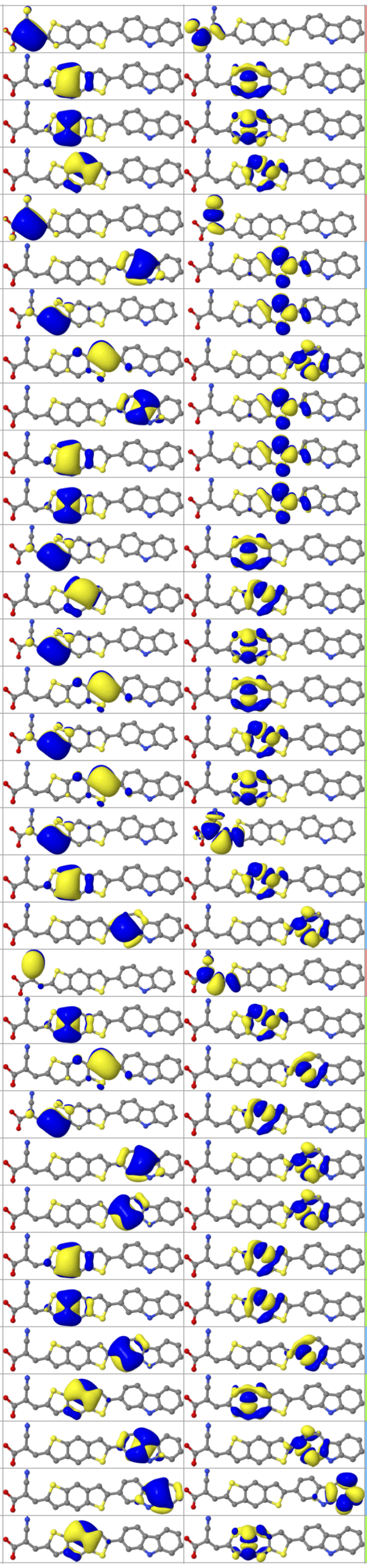

112	122	-0.09184	0.00843	A-pi	A-pi*
87	144	-0.09157	0.00839	B-pi	B-pi*
86	145	-0.08860	0.00785	B-pi	B-pi*
89	135	0.08317	0.00692	B-pi	B-pi*
112	119	-0.08156	0.00665	A-pi	A-pi*
83	118	-0.08049	0.00648	D-pi	B-pi*
113	118	0.07869	0.00619	B-pi	B-pi*
116	127	-0.07612	0.00579	B-pi	D-pi*
81	118	-0.07580	0.00575	D-pi	B-pi*
87	118	0.07507	0.00564	B-pi	B-pi*
86	118	-0.07396	0.00547	B-pi	B-pi*
113	144	-0.07106	0.00505	B-pi	B-pi*
85	146	0.07071	0.00500	B-pi	B-pi*
113	145	0.06979	0.00487	B-pi	B-pi*
116	144	-0.06964	0.00485	B-pi	B-pi*
113	135	-0.06897	0.00476	B-pi	B-pi*
116	145	0.06805	0.00463	B-pi	B-pi*
113	447	0.06751	0.00456	B-pi	A-pi*
87	135	-0.06751	0.00456	B-pi	B-pi*
82	127	-0.06738	0.00454	D-pi	D-pi*
114	121	-0.06627	0.00439	A-pi	A-pi*
86	135	0.06583	0.00433	B-pi	B-pi*
116	149	-0.06544	0.00428	B-pi	B-pi*
113	146	-0.06439	0.00415	B-pi	B-pi*
83	127	0.06386	0.00408	D-pi	D-pi*
94	127	-0.06376	0.00407	D-pi	D-pi*
87	146	-0.06233	0.00389	B-pi	B-pi*
86	146	0.06219	0.00387	B-pi	B-pi*
94	149	-0.06174	0.00381	D-pi	D-pi*
89	144	0.06083	0.00370	B-pi	B-pi*
81	127	0.05993	0.00359	D-pi	D-pi*
84	123	0.05816	0.00338	D-pi	D-pi*
89	145	-0.05813	0.00338	B-pi	B-pi*

Figure S2: Dominant contributions to the first excited state in benzodithiophene (CBA). The excitation goes from the initial to the final MO, whose isosurface and type are indicated in the last four columns. Only contributions with $|CI| > 0.05$ are shown. Donor MOs are labeled in blue, acceptor MOs in red, while green indicates MOs centered on the bridge.

**116**	**479**	-0.05722	0.00327	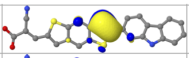	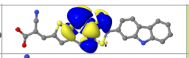	B-pi	B-pi*
**85**	**145**	-0.05613	0.00315	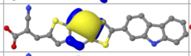	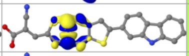	B-pi	B-pi*
**85**	**144**	0.05583	0.00312	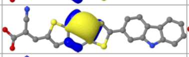	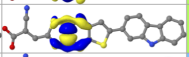	B-pi	B-pi*
**80**	**123**	0.05550	0.00308	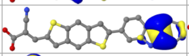	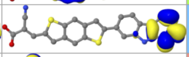	D-pi	D-pi*
**114**	**119**	-0.05509	0.00303	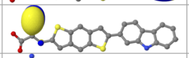	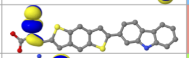	A-pi	A-pi*
**113**	**440**	0.05361	0.00287	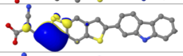	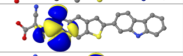	B-pi	B-pi*
**116**	**442**	0.05280	0.00279	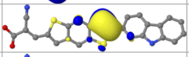	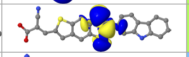	B-pi	B-pi*
**83**	**149**	0.05231	0.00274	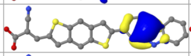	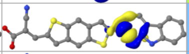	D-pi	D-pi*
**81**	**149**	0.05006	0.00251	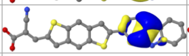	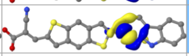	D-pi	D-pi*
			0.71466				
**B-pi**	**B-pi***		0.41571				
**D-pi**	**D-pi***		0.03179				
**A-pi**	**A-pi***		0.06086				
**B-pi**	**A-pi***		0.15859				
**A-pi**	**B-pi***		0.01067				
**B-pi**	**D-pi***		0.00579				
**D-pi**	**B-pi***		0.03123				
**SUM**			0.71466				
**CT**			0.20629				
**Directe**	**D->A**		0.18983				
**Directe**	**A->D**		0.01646				

Figure S3: Dominant contributions to the first excited state in benzodithiophene (CBA). The excitation goes from the initial to the final MO, whose isosurface and type are indicated in the last four columns. Only contributions with $|CI| > 0.05$ are shown. Donor MOs are labeled in blue, acceptor MOs in red, while green indicates MOs centered on the bridge. CT corresponds to the percentage of charge transfer excitations for the given threshold.

Initial	Final	CI	$\lvert CI \rvert^2$	Initial MO	Final MO	Initial MO type	Final MO type
109	117	-0.27980	0.07829	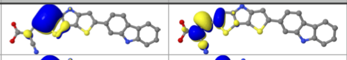		B-pi	A-pi*
109	118	-0.25327	0.06415	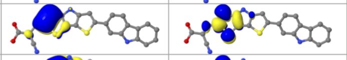		B-pi	B-pi*
87	118	0.22217	0.04936	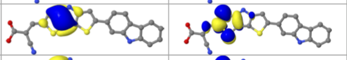		B-pi	B-pi*
85	118	-0.21862	0.04779	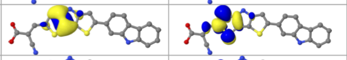		B-pi	B-pi*
110	117	-0.20815	0.04333	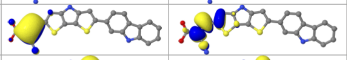		A-pi	A-pi*
112	115	0.18720	0.03504	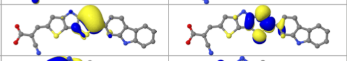		B-pi	B-pi*
87	117	0.17894	0.03202	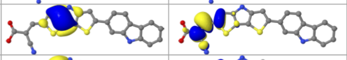		B-pi	A-pi*
85	117	-0.17597	0.03097	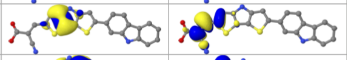		B-pi	A-pi*
88	118	-0.15188	0.02307	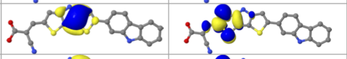		B-pi	B-pi*
86	118	0.14860	0.02208	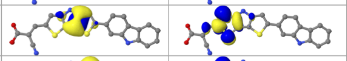		B-pi	B-pi*
112	118	-0.14483	0.02098	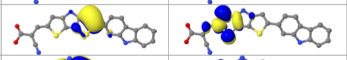		B-pi	B-pi*
88	117	-0.12831	0.01646	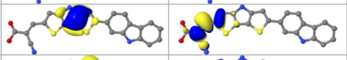		B-pi	A-pi*
112	117	-0.12546	0.01574	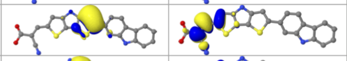		B-pi	A-pi*
86	117	0.12546	0.01574	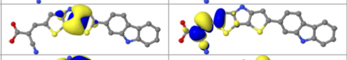		B-pi	A-pi*
88	115	0.12395	0.01536	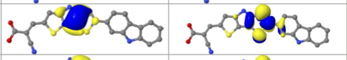		B-pi	B-pi*
86	115	-0.12133	0.01472	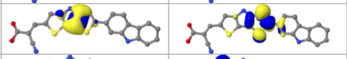		B-pi	B-pi*
110	118	-0.10436	0.01089	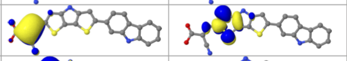		A-pi	B-pi*
109	120	-0.09950	0.00990	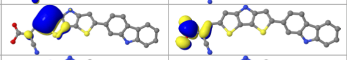		B-pi	A-pi*
110	120	-0.09913	0.00983	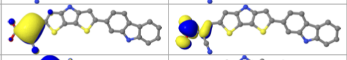		A-pi	A-pi*
109	116	0.09867	0.00974	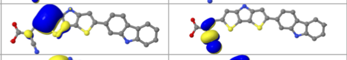		B-pi	A-pi*
85	130	0.09239	0.00854	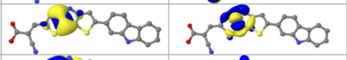		B-pi	B-pi*
87	139	-0.09190	0.00845	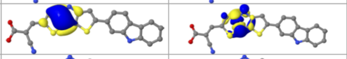		B-pi	B-pi*
110	116	0.09077	0.00824	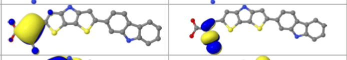		A-pi	A-pi*
87	115	-0.08596	0.00739	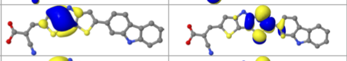		B-pi	B-pi*
85	115	0.08518	0.00726	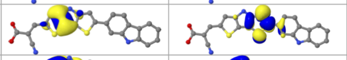		B-pi	B-pi*
87	130	-0.07945	0.00631	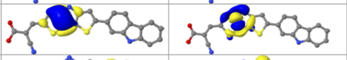		B-pi	B-pi*
93	115	0.07862	0.00618	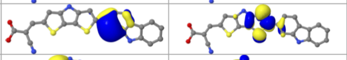		D-pi	B-pi*
85	139	0.07610	0.00579	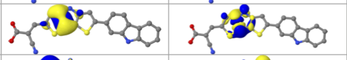		B-pi	B-pi*
109	115	0.07557	0.00571	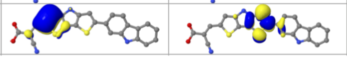		B-pi	B-pi*

Figure S4: Dominant contributions to the first excited state in dithienopyrrole (CDA). The excitation goes from the initial to the final MO, whose isosurface and type are indicated in the last four columns. Only contributions with $\lvert CI \rvert > 0.05$ are shown. Donor MOs are labeled in blue, acceptor MOs in red, while green indicates MOs centered on the bridge.

**88**	**140**	0.07390	0.00546	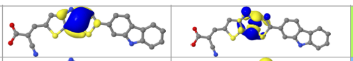		B-pi	B-pi*
**81**	**115**	-0.07352	0.00541	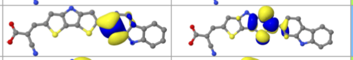		D-pi	B-pi*
**86**	**125**	0.07259	0.00527	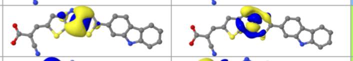		B-pi	B-pi*
**109**	**433**	-0.06901	0.00476	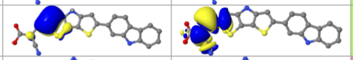		B-pi	A-pi*
**111**	**117**	-0.06719	0.00451	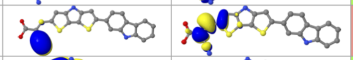		A-pi	A-pi*
**87**	**116**	-0.06677	0.00446	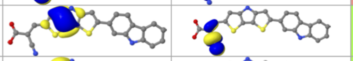		B-pi	A-pi*
**85**	**116**	0.06566	0.00431	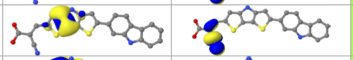		B-pi	A-pi*
**87**	**120**	0.06545	0.00428	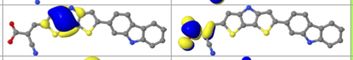		B-pi	A-pi*
**112**	**140**	0.06466	0.00418	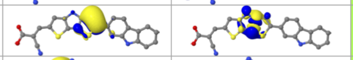		B-pi	B-pi*
**85**	**120**	-0.06437	0.00414	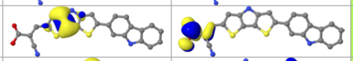		B-pi	A-pi*
**112**	**124**	0.06420	0.00412	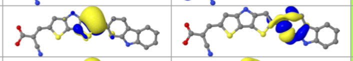		B-pi	D-pi*
**112**	**125**	-0.06402	0.00410	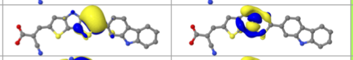		B-pi	B-pi*
**88**	**125**	-0.06141	0.00377	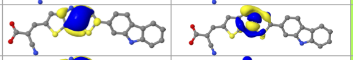		B-pi	B-pi*
**88**	**139**	0.06020	0.00362	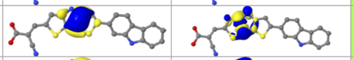		B-pi	B-pi*
**86**	**130**	-0.05993	0.00359	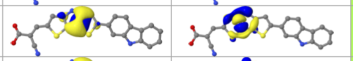		B-pi	B-pi*
**86**	**140**	-0.05942	0.00353	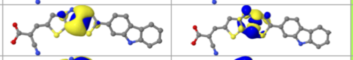		B-pi	B-pi*
**88**	**130**	0.05934	0.00352	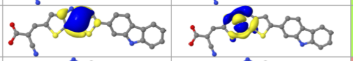		B-pi	B-pi*
**111**	**116**	0.05818	0.00338	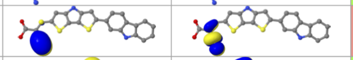		A-pi	A-pi*
**112**	**130**	0.05735	0.00329	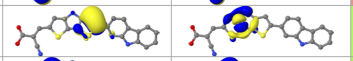		B-pi	B-pi*
**109**	**428**	-0.05720	0.00327	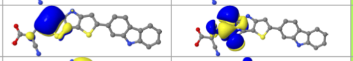		B-pi	B-pi*
**86**	**139**	-0.05713	0.00326	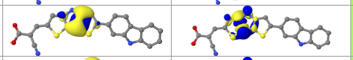		B-pi	B-pi*
**112**	**139**	0.05664	0.00321	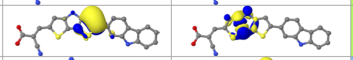		B-pi	B-pi*
**84**	**115**	-0.05624	0.00316	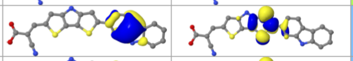		D-pi	B-pi*
**80**	**115**	0.05408	0.00292	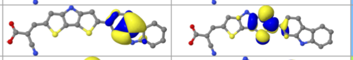		D-pi	B-pi*
**112**	**146**	-0.05355	0.00287	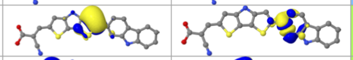		B-pi	D-pi*
**109**	**130**	0.05326	0.00284	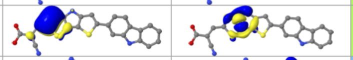		B-pi	B-pi*
**81**	**124**	-0.05309	0.00282	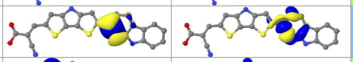		D-pi	D-pi*
**109**	**139**	0.05252	0.00276	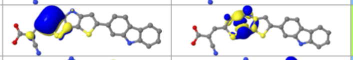		B-pi	B-pi*
**93**	**124**	0.05213	0.00272	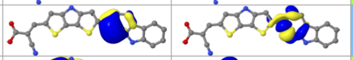		D-pi	D-pi*
**87**	**428**	0.05150	0.00265	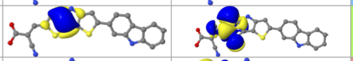		B-pi	B-pi*
**110**	**433**	-0.05075	0.00258	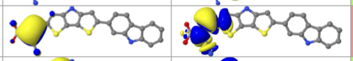		A-pi	A-pi*
**85**	**428**	-0.05058	0.00256	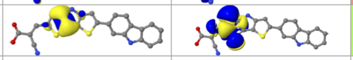		B-pi	B-pi*
**Sum**			0.74665				

Figure S5: Dominant contributions to the first excited state in dithienopyrrole (CDA). The excitation goes from the initial to the final MO, whose isosurface and type are indicated in the last four columns. Only contributions with $|CI| > 0.05$ are shown. Donor MOs are labeled in blue, acceptor MOs in red, while green indicates MOs centered on the bridge.

B-pi	B-pi*		0.40288				
D-pi	D-pi*		0.00554				
A-pi	A-pi*		0.07187				
D-pi	B-pi*		0.01767				
B-pi	A-pi*		0.23081				
A-pi	B-pi*		0.01089				
B-pi	D-pi*		0.00699				
			0.74665				
CT			0.26637				
Directec	D->A		0.24849				
Directec	A->D		0.01788				

Figure S6: Dominant contributions to the first excited state in dithienopyrrole (CDA). The excitation goes from the initial to the final MO, whose isosurface and type are indicated in the last four columns. Only contributions with $|CI| > 0.05$ are shown. Donor MOs are labeled in blue, acceptor MOs in red, while green indicates MOs centered on the bridge. CT corresponds to the percentage of charge transfer excitations for the given threshold.

S2 Computational details

All the RHF and pCCD-based calculations were performed in a developer version of the PyBEST software package.[1] All DFA calculations for CBA and CDA were done with the ADF2023[2,3] software package and the geometries from Ref. 4. In all pCCD-based calculations, a frozen core was applied, where the 1s orbitals of all C, N, and O atoms and the 1s, 2s, and 2p orbitals of S were frozen. All IP/EA-EOM-DLPNO-CCSD calculations were done with the ORCA 4.2.1 software package.[5–8]

References

[1] Boguslawski, K.; Leszczyk, A.; Nowak, A.; Brzęk, F.; Żuchowski, P. S.; Kędziera, D.; Tecmer, P. Pythonic Black-box Electronic Structure Tool (PyBEST). An open-source Python platform for electronic structure calculations at the interface between chemistry and physics. *Comput. Phys. Commun.* **2021**, *264*, 107933.

[2] te Velde, G.; Bickelhaupt, F. M.; Baerends, E. J.; Guerra, C. F.; van Gisbergen, S. J. A.; Snijders, J. G.; Ziegler, T. Chemistry with ADF. *J. Comput. Chem.* **2001**, *22*, 931–967.

[3] van Lenthe, E.; Baerends, E. J. Optimized Slater-type basis sets for the elements 1-118. *J. Comput. Chem.* **2003**, *24*, 1142–1156.

[4] Delgado-Montiel, T.; Baldenebro-López, J.; Soto-Rojo, R.; Glossman-Mitnik, D. Theoretical study of the effect of π-bridge on optical and electronic properties of carbazole-based sensitizers for DSSCs. *Molecules* **2020**, *25*, 3670.

[5] Neese, F. The ORCA program system. *WIREs Comput. Mol. Sci.* **2012**, *2*, 73–78.

[6] Neese, F. Software update: the ORCA program system, version 4.0. *WIREs Comput. Mol. Sci.* **2018**, *8*, e1327.

[7] Neese, F.; Wennmohs, F.; Becker, U.; Riplinger, C. The ORCA quantum chemistry program package. *J. Chem. Phys.* **2020**, *152*.

[8] Neese, F. Software update: The ORCA program system—Version 5.0. *WIREs Comput. Mol. Sci.* **2022**, *12*, e1606.
